# 

**Published:** 1904-08

**Authors:** 


### CONTRIBUTORS TO NEW SERIES VOLUME XLIV.

### (Whole Number Volume LX.)

### AUGUST, 1904—JULY, 1905.

---

Bainbridge, William S. .......... New York
Barrows, Franklin W. .......... Buffalo
Bayliss, A. W. .......... Buffalo
Benedict, A. L. .......... Buffalo
Bentz, Charles A. .......... Buffalo
Bishop, Mrs. J. F. .......... China
Brush, Edward N. .......... Towson, Md.
Burrows, Lorenzo, Jr. .......... Buffalo
Busch, Frederick C. .......... Buffalo
Chapin, Henry Dwight .......... New York
Clark, Edward .......... Buffalo
Clinton, Marshall .......... Buffalo
Cott, George F. .......... Buffalo
Crothers, T. D. .......... Hartford
Cumston, Charles Greene .......... Boston
DeWitt, C. .......... Washington
Edsall, D. L. .......... Philadelphia
Fisher, Edward D. .......... New York
Fowler, George B. .......... New York
Frazee, A. B. .......... Rochester
Frye, Maud J. .......... Buffalo
Gibson, James A. .......... Buffalo
Gram, Franklin C. .......... Buffalo
Grant, John H. .......... Buffalo
Greene, Walter D. .......... Buffalo
Creveling, J. P. .......... Auburn
Grosvenor, J. W. .......... Buffalo
Hanley, L. G. .......... Buffalo
Hopkins, Henry Reed .......... Buffalo
House, William .......... Portland, Ore
Houston, Sam .......... Washington
Hubbell, A. A. .......... Buffalo
Jacobson, Nathan .......... Syracuse
Johnson, Ray H. .......... Buffalo
Johnston, George Ben .......... Richmond
Joslin, Elliott P. .......... Boston
King, James E. .......... Buffalo

| | |
|---|---|
| Krauss, William C. | Buffalo |
| Lapp, Henry | Clarence |
| Le Seur, John W. | Batavia |
| Lewis, F. Park | Buffalo |
| Long, Eli H. | Buffalo |
| Love, Frank W. | Buffalo |
| Lytle, Albert T. | Buffalo |
| MacDonald, Carlos A. | New York |
| MacLeod, J. A. | Buffalo |
| McGuire, Edgar R. | Buffalo |
| McGuire, Francis W. | Buffalo |
| McKee, Thomas H. | Buffalo |
| Massey, Myrtle L. | Buffalo |
| Mewborn, A. D. | New York |
| Millener, F. H. | Buffalo |
| Morris, Robert T. | New York |
| Norton, Charles P. | Buffalo |
| Panzer, B. | Vienna |
| Park, Roswell | Buffalo |
| Phillips, William Linton | Buffalo |
| Potter, William Warren | Buffalo |
| Putnam, James Wright | Buffalo |
| Reed, Charles A. L. | Cincinnati |
| Renner, W. Scott | Buffalo |
| Ricketts, B. Merrill | Cincinnati |
| Rochester, DeLancey | Buffalo |
| Rowell, E. E. | Stamford |
| Ruggles, E. Wood | Rochester |
| Sawers, F. H. | Rochester |
| Schugens, M. Elizabeth | Buffalo |
| Sherman, Dewitt H. | Buffalo |
| Simmons, George H. | Chicago |
| Simpson, Burton T. | Buffalo |
| Snell, Albert C. | Rochester |
| Stephenson, Frank H. | Syracuse |
| Van der Beek, Charles A. | Rochester |
| Van Duzee, Edward P. | Buffalo |
| Van Fleet, Frank | New York |
| Wende, Grover W. | Buffalo |
| Wey, Hamilton D. | Elmira |
| Williams, Herbert U. | Buffalo |
| Wilson, Nelson W. | Buffalo |

Alumni Association, University of Buffalo.
Buffalo Academy of Medicine.
Medical Association of Central New York.
Medical Society of the County of Erie.
Roswell Park Medical Club.

### BOOKS RECEIVED.

International Clinics. A Quarterly of Illustrated Clinical Lectures and especially prepared original articles on Treatment, Medicine, Surgery, Neurology, Pediatrics, Obstetrics, Gynecology, Orthopedics, Pathology, Dermatology, Ophthalmology, Otology, Rhinology, Laryngology, Hygiene and other topics of interest to students and practitioners. By leading members of the medical profession throughout the world. Edited by A. O. J. Kelly, A.M., M.D., Philadelphia. Volume II. Fourteenth series. 1904. Philadelphia: J. B. Lippincott Company. (Cloth, $2.00.)

An Introduction to Vertebrate Embryology, based on the Study of the Frog and the Chick. By Albert Moore Reese, Ph.D., Associate Professor of Histology in Syracuse University. Duodecimo, pp. 291. Illustrated. New York and London: G. P. Putnam's Sons. 1904.

A System of Practical Surgery. By Drs. E. von Bergmann, Berlin, P. von Bruns, Tübinger and J. von Mikulicz, Breslau. Translated and edited by William T. Bull, M.D., Professor of Surgery in the College of Physicians and Surgeons (Columbia University), New York, and John B. Solley, M.D., New York. To be complete in five imperial octavo volumes. Volume III. Surgery of the Extremities. Philadelphia and New York: Lea Brothers & Company. 1904. (Price, per volume: cloth, $6.00; leather, $7.00; half morocco, $8.50.)

The Gazette Pocket Speller and Definer. English and Medical. Second edition. New York: The Gazette Publishing Company. 1904. (Price, 50 cents.)

The Surgery of the Heart and Lungs. By Benjamin Merrill Ricketts, Ph.B., M.D., Cincinnati. Octavo, pp. 526. Illustrated. New York: The Grafton Press. 1904.

A Reference Handbook of the Medical Sciences. Embracing the Entire Range of Scientific and Practical Medicine and Allied Science. By Various Writers. A new edition, completely revised and rewritten. Edited by Albert H. Buck, M.D., New York City. Nine volumes, imperial octavo. Volume VIII. Illustrated by chromolithographs and 435 half-tone and wood engravings. New York: William Wood & Co. 1904. (Price: muslin, $6.00 per volume; leather, $7.00 per volume; half morocco, $8.00 per volume.)

A Textbook of Mechano-Therapy (Massage and Medical Gymnastics) for Medical Students, Trained Nurses and Medical Gymnasts. By Axel V. Grafstrom, B.Sc., M.D., Attending Physician to the Gustavus Adolphus Orphanage, Jamestown, N. Y. Second edition, revised, enlarged, and entirely reset. Duodecimo of 200 pages, fully illustrated. Philadelphia, New York, London: W. B. Saunders & Company. 1904. (Cloth, $1.25 net.)

